# Reactive attachment disorder and disinhibited social engagement disorder in adolescence: co-occurring psychopathology and psychosocial problems

**DOI:** 10.1007/s00787-020-01673-7

**Published:** 2020-11-13

**Authors:** Astrid R. Seim, Thomas Jozefiak, Lars Wichstrøm, Stian Lydersen, Nanna S. Kayed

**Affiliations:** 1 Department of Child and Adolescent Psychiatry, St. Olavs Hospital, Trondheim, Norway; 2 Department of Mental Health, Faculty of Medicine and Health Sciences, NTNU Norwegian University of Science and Technology, Trondheim, Norway; 3 Department of Psychology, NTNU, Trondheim, Norway

**Keywords:** Adolescence, Child welfare, Comorbidity, Disinhibited social engagement disorder, Mental health, Psychosocial problems, Reactive attachment disorder

## Abstract

Insufficient care is associated with most psychiatric disorders and psychosocial problems, and is part of the etiology of reactive attachment disorder (RAD) and disinhibited social engagement disorder (DSED). To minimize the risk of misdiagnosis, and aid treatment and care, clinicians need to know to which degree RAD and DSED co-occur with other psychopathology and psychosocial problems, a topic little researched in adolescence. In a national study of all adolescents (*N* = 381; 67% consent; 12–20 years old; 58% girls) in Norwegian residential youth care, the Child and Adolescent Psychiatric Assessment interview yielded information about psychiatric diagnoses and psychosocial problems categorized as present/absent, and the Child Behavior Check List questionnaire was applied for dimensional measures of psychopathology. Most adolescents with a RAD or DSED diagnosis had several cooccurring psychiatric disorders and psychosocial problems. Prevalence rates of both emotional and behavioral disorders were high in adolescent RAD and DSED, as were rates of suicidality, self-harm, victimization from bullying, contact with police, risky sexual behavior and alcohol or drug misuse. Although categorical measures of co-occurring disorders and psychosocial problems revealed few and weak associations with RAD and DSED, dimensional measures uncovered associations between both emotional and behavioral problems and RAD/DSED symptom loads, as well as DSED diagnosis. Given the high degree of comorbidity, adolescents with RAD or DSED—or symptoms thereof—should be assessed for co-occurring psychopathology and related psychosocial problems. Treatment plans should be adjusted accordingly.

**Electronic supplementary material:**

The online version of this article (10.1007/s00787-020-01673-7) contains supplementary material, which is available to authorized users.

## Introduction

Childhood maltreatment and neglect are associated with a wide range of psychiatric disorders and psychosocial problems [1–3] and may, in severe cases, cause reactive attachment disorder (RAD) and disinhibited social engagement disorder (DSED) [4]. Distinguishing between various health effects of childhood adversity may be clinically challenging, and there is a particular risk and tendency to misdiagnose RAD and DSED [5, 6], either by the under-identification of common psychiatric disorders and neurodevelopmental problems and the over-identification of attachment problems [5, 7–9] or vice versa [10, 11]. Misdiagnosis may result in missed treatment and developmental support, prolonging individual suffering, functional impairment, and societal costs. To improve diagnostic precision and aid the development of appropriate treatment plans, health and social workers need to be knowledgeable about the degree of co-occurrence between RAD or DSED and other psychopathology and psychosocial problems. However, at present, we know comparatively little about this co-occurrence in adolescence. Although several studies have investigated the associations between RAD and DSED and other psychopathology in preschool and school-aged children, existing results are inconsistent and may be prone to type-II error due to categorical approaches and small sample sizes [12, 13]. Moreover, because of heterotypic continuity and differences in rates of psychopathology between childhood and adolescence [2, 14, 15], findings in younger children cannot necessarily be ascribed to adolescents. The psychiatric comorbidities of RAD and DSED may also differ in different contexts, and remain largely unexplored among adolescents exposed to in-family maltreatment and neglect where placement in well-functioning foster or adoptive families has failed, thus culminating in institutional care. Furthermore, additional psychosocial problems known to be associated with maltreatment and neglect, including suicidality, self-harm, alcohol or drug misuse, victimization from bullying, risky sexual behavior and criminal behavior [1, 3], may influence care and treatment for children with RAD or DSED. At present, we do not know the extent to which such problems are present in RAD and DSED. Therefore, to help clinicians and services attend to the complex clinical picture often presented in individuals exposed to early adversity, while having access to a national high-risk sample, we investigate the rates of co-occurrence and strengths of associations between RAD and DSED and other psychiatric disorders and emotional, behavioral and psychosocial problems among adolescents living in residential youth care (RYC).

RAD is characterized by social withdrawal and aberrant attachment behavior with failure to seek and respond to comfort, whereas DSED is characterized by socially disinhibited behavior and the lack of reticence in unfamiliar settings or interactions with strangers [4]. Both RAD and DSED have been demonstrated as valid diagnostic constructs, distinct from other psychopathology in childhood and adolescence [16–22], and symptoms may persist into adolescence and early adulthood, with potentially large individual and societal costs [2, 23–26]. Because the fourth edition of the Diagnostic and Statistical Manual of Mental Disorders (DSM-IV) defines the concepts corresponding with RAD and DSED as two subtypes of one disorder [27], contrary to the two distinct disorders in the DSM-5 [4], previous research often investigated RAD and DSED as a combined diagnostic concept. A combined RAD and DSED is reported to frequently co-occur with both emotional symptoms and disorders (such as depression and anxiety) and behavioral symptoms and disorders (such as oppositional defiant disorder (ODD), conduct disorder (CD) or attention deficit hyperactive disorder (ADHD)) [28–37]. When distinguishing between RAD and DSED, scientists have expected RAD (or the ‘RAD inhibited type’ in studies using the DSM-IV), with its socially withdrawn phenotype, to be associated with emotional problems and DSED (or the ‘RAD disinhibited type’ in studies using the DSM-IV), with its indiscriminate phenotype, to be associated with behavioral problems [22].

Some studies on RAD have confirmed these expectations in preschool and school-aged children [13, 38, 39]. Others have found that symptoms of RAD in preschoolers have no correlations with any psychopathology [36], or found ambiguous associations depending on the sample [40]. Contrary are findings that RAD symptoms or disorder in school-age and early adolescence may be associated with both emotional and behavioral problems [26, 41–43]. Beyond age 12, the comorbidity for RAD remains unstudied.

Similarly, for DSED in preschool and school-aged children, some studies have confirmed the above expectations, either by investigating associations with behavioral and not emotional problems [11, 19] or by investigating both and finding associations only with behavioral problems [41, 44, 45]. Conversely, others have found that DSED in preschool and school-age children may co-occur [31, 38, 42] or be positively associated [36, 39, 46] with both emotional and behavioral disorders or problems. Beyond school age, persistent DSED symptoms in young adults adopted from early institutional deprivation to well-functioning families in preschool age have been found to be associated with symptoms of ADHD and callous-unemotional traits (CU), though unrelated to depression, anxiety and CD symptoms [47]. Of note, generalizability to populations unexposed to early severe deprivation in institutions has been questioned [2, 48]. Early institutionalization is no longer a common practice in industrialized countries [46]. However, exposure to in-family maltreatment and neglect remains a major public health concern [1, 49]. In non-institutionalized adolescents exposed to in-family maltreatment, DSED symptoms have been found to be associated with both emotional and behavioral problems [46], but prevalence rates of co-occurring psychiatric disorders and psychosocial problems remain undescribed.

To allow for advantages regarding both categorical and dimensional approaches to psychopathology [50], we use four approaches to illuminate the co-occurrence between RAD and DSED and other psychopathology and psychosocial problems. First, we investigate the degree to which other psychiatric disorders and categorical psychosocial problems (categorized as present or absent) co-occur with RAD and DSED diagnoses. This approach may be of clinical value, as diagnoses are clinical tools comprising more than mere cut-off values of symptom loads (i.e., taking into account onset, duration, distress, impairment and exclusion criteria). Second, we investigate whether the risks of having co-occurring psychiatric disorders and categorical psychosocial problems change with increasing RAD or DSED symptom loads. This approach affords higher statistical power than treating RAD and DSED as dichotomous variables and allows the inclusion of cases with sub-threshold levels of RAD and DSED symptom loads, where the psychiatric burden and impairment may be high despite the unfulfillment of some diagnostic criteria [50]. Third, inversely, as individuals with RAD or DSED may also be debilitated by other sub-threshold psychopathology, we investigate the levels of dimensionally measured emotional and behavioral problems for adolescents with RAD and DSED diagnoses compared to those without. Finally, we apply a dimensional approach to all variables and investigate whether emotional and behavioral problems are associated with RAD or DSED symptom loads. This final approach further increases statistical power and allows the analysis of sub-threshold cases with respect to both co-occurring psychopathology and RAD/DSED. Because RAD and DSED are distinct disorders in adolescence [21, 24], they are investigated separately in each approach.

In sum, we aim to study the rates of co-occurrence and strengths of associations between RAD and DSED, respectively, and other psychopathology and psychosocial problems in adolescence. We do so by assessing high-risk adolescents living in Norwegian RYC using in-depth psychiatric interviews and investigate psychiatric disorders and psychosocial problems (categorized as present or absent) and their (1) prevalence and odds in adolescents with RAD and DSED diagnoses; (2) association with increasing RAD and DSED symptom loads. Further, using a dimensional approach to other psychopathology, we investigate (3) the levels of emotional and behavioral problems in adolescents with RAD and DSED diagnoses; (4) whether emotional and behavioral problems are associated with RAD and DSED symptom loads.

## Methods

### Participants

The research project Mental Health in Adolescent Residents in the Child Welfare System [51] invited all residents aged 12–23 years living in Norwegian RYC between 2011 and 2014 to participate. Due to a presumed state of high crisis, adolescents in acute placements and unaccompanied minors without Norwegian asylum were excluded, as were adolescents without sufficient Norwegian language proficiency to complete the psychiatric interviews. In total, 400 of 601 (67%) eligible adolescents in 86 RYC institutions consented, with *N *= 381 yielding information about RAD and DSED. The recruitment flowchart is shown in Figure S1 (Online Resource). The participants were between 12.2 and 20.2 years old (*M* = 16.7, SD 1.4), 57.7% were girls (*n *= 220), and 78.2% were ethnic Norwegian. The mean age at the first out-of-home placement was 12.5 years (SD 3.9), and the mean number of out-of-home placements was 3.3 (SD 2.4). In total, *n *= 8 participants had previously been diagnosed with mild intellectual disability, whereof *n *= 1 qualified for RAD and *n *= 2 for DSED. Previous studies of the same participants revealed very high rates of psychiatric morbidity and high levels of parental risk factors, such as drug use or mental or chronic illness [51]. Virtually, all the participants were likely exposed to in-family neglect, and 71% self-reported exposure to maltreatment [52]. We have previously reported the symptom frequency range for RAD and DSED as 2–35 and 4–11%, respectively, and the diagnose prevalence rates as 9% RAD (*n *= 33) and 8% DSED (*n *= 31), with 0.5% (*n *= 2) having both disorders [21].

### Setting

The primary aim of the Norwegian child protection services (CPS) is to provide in-family support to children and families in need and invoke out-of-home placements only when considered necessary to secure provision of a child’s basic needs [53]. In such cases, foster care is preferred, and RYC represents a last resort [54]. In accordance with the CPS criteria for out-of-home placements [55], adolescents living in Norwegian RYC have likely been exposed to social neglect, inadequate care or maltreatment prior to placement. Although placements due to behavioral problems or drug use are more frequent for adolescents in RYC than in foster care, traits of the caregiving environment (e.g., parental mental illness or drug use, lack of caregiving ability or other factors in the home) are the most common reasons for placement, regardless of placement type [54].

Norwegian RYC institutions typically resemble family homes with three–eight residents and are strictly regulated by law and quality requirements to ensure that all residents are provided with basic needs and a secure, developmentally supportive environment [56]. There is awareness of the importance of relational continuity. Every resident has a designated primary contact whose aims are to establish a trusting relationship and fulfill the role of a primary caretaker for their designated resident. Given these circumstances, and the fact that 90% of the participants reported to have lived at least three months in RYC prior to the data collection [57], the primary contacts were trusted as reliable informants. Further details on the setting are given in [21, 51].

### Procedure

The data were collected at RYC institutions from June 2011 to July 2014. Four trained research assistants with relevant professional backgrounds completed semi-structured psychiatric interviews with the participants and their primary contacts. The study was approved by the Norwegian Regional Committee for Medical and Health Research Ethics, REC central Norway, and all participants gave written informed consent.

### Measures

#### Interview with adolescents

The Child and Adolescent Psychiatric Assessment (CAPA) [58] is an in-depth semi-structured psychiatric interview which determines psychiatric disorders in children and adolescents, as defined by DSM-IV. The CAPA collects information about symptom onset, duration, frequency and intensity and includes both required and optional follow-up questions. Interviewers probe until they clarify the presence of predefined symptom criteria. The following psychiatric disorder categories and psychosocial problems were assessed using CAPA: depression, anxiety, CD/ODD, suicidal thoughts, suicidal plan, suicidal attempt, suicidal behavior without suicidal intent, self-injurious behavior without suicidal intent (self-harm), exposure to bullying, contact with police, sex for gain, substance use (daily use of alcohol or ever having used cannabis or hard drugs) and substance use for mood improvement. A three-month primary period was applied to all the CAPA variables, except for the following, where a lifetime period was applied: suicidal attempt, been bullied often, contact with police, sex for gain and substance use.

#### Interview with the adolescents’ primary contacts

Adolescents are considered to be less reliable informants regarding symptoms of ADHD than adults who know them well [59]. Further, because self-acknowledging signs of RAD and DSED would require mentalization abilities beyond what could be expected of adolescents with RAD and DSED, due to the lack of supportive caregiving relationships necessary to promote mentalization [60], adolescents were expected to be sub-optimal informants of RAD and DSED symptoms. Therefore, ADHD, RAD and DSED were assessed using the adolescents’ primary contacts as informers. ADHD was assessed using the caregiver version of CAPA and RAD/DSED using the RAD module in the Preschool Age Psychiatric Assessment (PAPA) [61]. The DSM-5 criteria [4] were applied in diagnosing RAD and DSED; however, we lacked the RAD item ‘response to comfort’.

To prevent interviewer drift and ensure adherence to the interview protocol, the interviews underwent regular and random controls. To provide inter-rater reliability estimates, blinded raters re-coded a randomly drawn sample (*n *= 42; 10.5%) of interview audio recordings. Inter-rater reliability for the DSM-IV by Gwet’s AC_1_ was in the range of 0.74–1.0, and the absolute agreement was in the range of 83–100% [51].

#### Child Behavior Checklist (CBCL)

To obtain information about sub-threshold emotional and behavioral problems, the adolescents’ primary contacts completed the CBCL for ages 6–18 [62], a well-validated caregiver questionnaire with 118 items, yielding the following syndrome scales: anxiety/depressed, withdrawn/depressed, somatic complaints, social problems, thought problems, attention problems, rule-breaking behavior and aggressive behavior. The CBCL items classified as ‘other problems’ were also included.

### Statistical analysis

Among the 381 subjects, 59 cases had information about RAD, DSED, ADHD, and CBCL syndrome scales, but had missing information about other CAPA-informed comorbid disorders and psychosocial problems because their primary contacts had completed the diagnostic interview, but the adolescents themselves had not. Missing data were handled by multiple imputation. In the imputation model, we used all variables to be included in the analysis. Imputation for girls and boys was done separately. We created 100 imputed data sets, generally regarded as sufficient [63]. We chose not to restrict the imputed values to the possible range, as recommended by Rodwell et al. [64]. Differences in means were analyzed using the Student’s *t* test. Associations between RAD or DSED and the continuous variables were investigated using linear regression and the dichotomous variables using logistic regression. All regression analyses were adjusted for age and gender. Neither age at first placement nor the number of out-of-home placements were in complete case analyses associated with RAD or DSED diagnosis or symptom loads, and were not included in the imputation model. Two-sided *p* values < .05 were taken to indicate statistical significance, and 95% confidence intervals (CI) are reported where relevant. Due to multiple hypotheses, *p* values between .01 and .05 should be interpreted with caution. We used SPSS 25 for all analyses.

## Results

### RAD

**Fig. 1:**
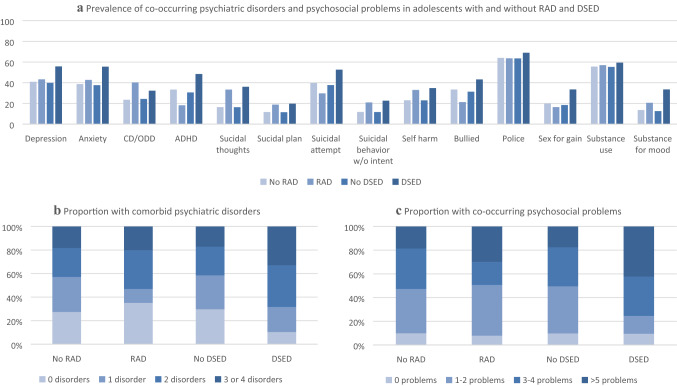
Prevalence (%) of co-occurring psychiatric disorders and psychosocial problems in adolescents with and without RAD and DSED diagnosis (**a**). Proportion (%) of adolescents with and without a RAD and DSED diagnosis who have co-occurring psychiatric disorders (**b**) and psychosocial problems (**c**)

Among adolescents with a RAD diagnosis, all disorders (Table 1, Fig. 1) were prevalent, and 65% fulfilled the criteria for at least one additional psychiatric disorder, with 53% fulfilling the criteria for at least two and 20% at least three. Further, all categorical psychosocial problems (Table 1, Fig. 1) were prevalent among adolescents with a RAD diagnosis, and 92% reported at least one co-occurring psychosocial problem, with 49% reporting at least three and 30% at least five. Nevertheless, this high-risk sample presented no differences in the rates of categorical psychiatric disorders or psychosocial problems for adolescents with a RAD diagnosis, compared to those without, except for suicidal thoughts, for which adolescents with a RAD diagnosis had 2.5 times increased odds (Table 1). Adolescents with a RAD diagnosis had means of 1.44 comorbid psychiatric disorders (range 0–4, mean difference 0.08 (CI − 0.35 to 0.51, *p *= .72) higher than adolescents without RAD) and 3.15 co-occurring psychosocial problems (range 0–10, mean difference 0.26 (CI − 0.50 to 1.02, *p *= .51) higher than adolescents without RAD). The odds of depression and anxiety increased with an increasing number of RAD symptoms, as did the odds of self-harm (Table 1). The remaining psychiatric disorders and categorical psychosocial problems were not associated with RAD symptom load in this high-risk sample. Through dimensional measures of other psychopathology, the sole clinically significant regression coefficient and statistically significant association for a RAD diagnosis was with the CBCL withdrawn/depressed syndrome scale (Table 1). However, the RAD symptom load had clinically significant regression coefficients and statistically significant associations with all the CBCL syndrome scales, except rule-breaking behavior (Table 1).

**Table 1:** RAD and DSED diagnosis and symptom load in adolescence: (a) prevalence and Odds Ratio (OR) for co-occurring psychiatric disorders and psychosocial problems; (b) association with Child Behavior Checklist (CBCL) syndrome scales

(a) Disorder/Psychosocial problem	Total *N *= 381	RAD diagnosis *n *= 33					RAD symptom load Range 0–11			DSED diagnosis *n *= 31					DSED symptom load Range 0–4		
	*n*	*n*	%	OR	CI	*p*	OR	CI	*p*	*n*	%	OR	CI	*p*	OR	CI	*p*
Depression	156.8	14.3	43.3	1.08	0.47 to 2.47	.85	**1.13**	**1.00 to 1.28**	**.043**	17.3	55.8	1.47	0.66 to 3.26	.35	1.18	0.85 to 1.65	.32
Anxiety	148.8	14.1	42.7	1.18	0.52 to 2.69	.70	**1.17**	**1.04 to 1.31**	**.009**	17.2	55.5	1.93	0.87 to 4.26	.10	1.33	0.96 to 1.86	.088
CD/ODD	95.1	13.3	40.3	2.22	0.95 to 5.19	.066	1.03	0.90 to 1.17	.72	10.0	32.3	1.89	0.75 to 4.75	.18	1.40	0.95 to 2.07	.091
ADHD	122	6	18.2	0.45	0.18 to 1.12	.084	1.02	0.91 to 1.14	.79	15	48.4	**2.50**	**1.17 to 5.36**	**.018**	**1.14**	**1.02 to 1.93**	**.035**
Any disorder	274.0	21.4	64.8	0.68	0.31 to 1.48	.33	1.13	0.99 to 1.29	.064	27.9	90.0	**3.48**	**1.02 to 11.86**	**.046**	**1.89**	**1.17 to 3.05**	**.010**
Suicidal thoughts	68.3	11.0	33.3	**2.51**	**1.00 to 6.29**	**.049**	1.09	0.94 to 1.27	.27	11.2	36.1	**2.49**	**1.01 to 6.13**	**.047**	**1.53**	**1.03 to 2.27**	**.037**
Suicidal plan	46.5	6.2	18.8	1.69	0.50 to 5.69	.40	1.11	0.92 to 1.35	.27	6.1	19.7	1.61	0.44 to 5.83	.47	1.35	0.78 to 2.33	.28
Suicidal attempt	148.2	9.8	29.7	0.65	0.27 to 1.57	.34	1.02	0.90 to 1.15	.80	16.3	52.6	1.68	0.74 to 3.80	.21	1.19	0.85 to 1.67	.31
Suic.beh w/o intent	47.6	6.9	20.9	1.90	0.54 to 6.67	.32	1.07	0.88 to 1.30	.48	7.0	22.6	2.26	0.67 to 7.68	.19	1.22	0.70 to 2.13	.48
Self-harm	91.0	10.9	33.0	1.60	0.65 to 3.96	.31	**1.16**	**1.00 to 1.33**	**.047**	10.8	34.8	1.27	0.52 to 3.10	.60	1.17	0.80 to 1.69	.42
Been bullied often	123.1	7.0	21.2	0.51	0.21 to 1.23	.14	1.04	0.93 to 1.17	.51	13.4	43.2	1.33	0.61 to 2.88	.48	1.20	0.88 to 1.65	.25
Contact with police	243.6	21.0	63.6	1.04	0.44 to 2.47	.94	0.99	0.88 to 1.12	.85	21.4	69.0	1.49	0.62 to 3.62	.38	1.20	0.83 to 1.74	.33
Sex for gain	74.7	5.4	16.4	0.69	0.15 to 3.22	.63	1.06	0.89 to 1.26	.54	10.4	33.5	2.14	0.76 to 6.01	.15	1.36	0.83 to 2.21	.22
Substance use	212.1	18.8	57.0	1.15	0.51 to 2.61	.74	0.97	0.87 to 1.09	.64	18.4	59.4	1.35	0.59 to 3.07	.48	1.21	0.85 to 1.72	.28
Substance for mood	54.3	6.8	20.6	1.61	0.50 to 5.14	.42	1.03	0.85 to 1.25	.74	10.4	33.5	**3.80**	**1.23 to 11.70**	**.020**	1.63	0.99 to 2.70	.055

(b) CBCL scale	RAD diagnosis				RAD symptom load (0–11)			DSED diagnosis						DSED symptom load (0-4)			
	*M* (S.E.)	*β*	CI	*p*	*β*	CI	*p*	M (S.E.)		*β*		CI	*p*	*β*	CI		*p*
Anxiety/depressed	6.91 (1.05)	0.41	− 1.35 to 2.16	.65	**0.65**	**0.40 to 0.90**	**<** **.001**	8.96 (1.08)		**2.25**		**0.41 to 4.09**	**.016**	**1.20**	**0.45 to 1.95**		**.002**
Withdrawn/depressed	6.73 (0.63)	**2.37**	**1.25 to 3.49**	**< .001**	**0.73**	**0.58 to 0.89**	**< .001**	4.75 (0.61)		0.11		− 1.12 to 1.34	.86	0.21	− 0.29 to 0.71		.42
Somatic complaints	4.00 (0.72)	0.00	− 1.36 to 1.36	1.0	**0.22**	**0.02 to 0.42**	**.031**	4.72 (0.80)		0.30		− 1.12 to 1.72	.68	0.18	− 0.41 to 0.77		.54
Social problems	4.55 (0.55)	− 0.21	− 1.52 to 1.11	.76	**0.37**	**0.18 to 0.57**	**<** **.001**	7.32 (0.86)		**2.71**		**1.33 to 4.09**	**< .001**	**1.56**	**1.01 to 2.12**		**<** **.001**
Thought problems	4.09 (0.62)	− 0.31	− 1.63 to 1.01	.64	**0.43**	**0.24 to 0.62**	**<** **.001**	6.33 (0.78)		**2.34**		**0.95 to 3.72**	**.001**	**1.23**	**0.68 to 1.79**		**<** **.001**
Attention problems	6.79 (0.70)	− 0.64	− 2.08 to 0.78	.37	**0.40**	**0.20 to 0.61**	**<** **.001**	10.35 (0.72)		**3.40**		**1.94 to 4.87**	**< .001**	**1.81**	**1.22 to 2.39**		**<** **.001**
Rule-breaking behavior	9.67 (1.00)	0.36	− 1.76 to 2.47	.74	0.11	− 0.20 to 0.43	.48	13.17 (1.22)		**4.17**		**1.95 to 6.39**	**< .001**	**2.47**	**1.57 to 3.36**		**<** **.001**
Aggressive behavior	10.67 (1.25)	0.18	− 2.49 to 2.86	.89	**0.57**	**0.18 to 0.97**	**.004**	16.14 (1.54)		**5.88**		**3.08 to 8.68**	**< .001**	**3.06**	**1.92 to 4.20**		**<** **.001**
Other problems	3.88 (0.51)	− 0.62	− 1.77 to 0.53	.29	**0.26**	**0.09 to 0.43**	**.003**	6.12 (0.73)		**1.94**		**0.73 to 3.16**	**.002**	**1.12**	**0.62 to 1.61**		**<** **.001**

Reference group: adolescents without RAD or DSED, respectively. All analyses are adjusted for age and gender. 1a) Logistic regression analyses with the comorbid disorder or psychosocial problem as dependent variable, RAD/DSED diagnosis or symptom load as covariate. Analyses and estimated ‘*n*’ with decimals are based on multiple imputation. 1b) Linear regression with RAD/DSED diagnosis or symptoms as covariates

*β* unstandardized regression coefficient, *M* estimated mean, *S.E.* standard error, *ADHD* attention deficit hyperactive disorder, *CD* conduct disorder, *DSED* disinhibited social engagement disorder, *ODD* oppositional defiant disorder, *RAD* reactive attachment disorder, *Substance use* daily alcohol use or ever having used cannabis or hard drugs, *Suic.beh w/o intent* suicidal behavior without suicidal intention

### DSED

All disorders (Table 1, Fig. 1) were prevalent among adolescents with a DSED diagnosis, and 90% fulfilled the criteria for at least one additional psychiatric disorder, with 68% fulfilling the criteria for at least two and 33% at least three. The odds of ADHD were 2.5 times higher, and the odds of any other psychiatric disorder 3.5 times higher for adolescents with a DSED diagnosis than those without. Adolescents with a DSED diagnosis had a mean of 1.92 comorbid disorders (range 0–4, mean difference 0.60 (CI 0.17 to 1.03, *p *= .006) higher than adolescents without DSED). All categorical psychosocial problems were also prevalent among adolescents with a DSED diagnosis (Table 1, Fig. 1), and 91% reported at least one co-occurring psychosocial problem, with 76% reporting at least three and 42% at least five. Adolescents with a DSED diagnosis had a mean of 4.04 co-occurring psychosocial problems (range 0–10, mean difference 1.23 (CI 0.34 to 2.11, *p *= .006) higher than adolescents without DSED). The odds of suicidal thoughts and substance use for mood improvement were higher among adolescents with a DSED diagnosis compared to those without. Further, the odds of having ADHD, any comorbid disorder or suicidal thoughts increased with increasing DSED symptom load (Table 1). For the remaining categorical psychiatric disorders and psychosocial problems, we found no associations with DSED symptom load in this high-risk sample. However, by dimensional measures of other psychopathology, we found DSED diagnosis and symptom load to be associated with the following CBCL syndrome scales: anxiety/depressed, social problems, thought problems, attention problems, rule-breaking behavior, aggressive behavior and other problems (Table 1). Scores on the CBCL syndrome scales withdrawn/depressed and somatic complaints were neither associated with DSED diagnosis nor symptom load.

## Discussion

RAD and DSED are often misdiagnosed in children and adolescents with histories of neglect, either by overidentifying or overlooking the attachment-related nature of their problems or—when rightly recognized—not acknowledging comorbid conditions. To counteract this tendency toward misdiagnosis and elaborate on the complex clinical picture often presented in individuals exposed to early adversity, we investigated the co-occurrence of other psychopathology and psychosocial problems among adolescents with and without RAD and DSED in a national study of high-risk adolescents living in RYC. This is the first in-depth study of RAD and DSED comorbidity in adolescence to report prevalence rates of co-occurring psychiatric disorders and psychosocial problems, and to explore associations using both categorical and dimensional approaches to psychopathology. We found that most adolescents with RAD or DSED diagnoses had additional psychiatric disorders and psychosocial problems and that all investigated disorders and psychosocial problems of both emotional and behavioral types frequently co-occurred with both RAD and DSED. In general, analyses based on categorical variables have lower statistical power than those based on the corresponding scale variables. Indeed, in analyses of associations between RAD and DSED and other psychopathology in this high-risk sample, the choice of categorical measures versus dimensional measures was decisive for the results. The categorical measures in the analytical approaches to co-occurring psychopathology and psychosocial problems revealed few and not highly statistically significant differences between adolescents with and without RAD and DSED, whereas the dimensional measures clearly showed that RAD and DSED symptom loads and a DSED diagnosis were all associated with both emotional and behavioral problems.

Consonant with the lasting negative effects of childhood neglect and maltreatment [1, 65], the prevalence rates of both emotional and behavioral disorders [14, 66, 67] and frequencies of psychosocial problems [68–70] were markedly higher among adolescents with RAD or DSED than in the general population and were comparable to findings in other adolescents subjected to child abuse and neglect [71]. The prevalence rates were as high or higher than in early institutionalized preschool and 12-year-old children assigned to ‘care as usual’ in the Bucharest Early Intervention Project (BEIP) [72, 73]. Possibly, individuals with RAD/DSED and co-occurring psychopathology or psychosocial problems have increased risk of repeated placement breakdown with subsequent placement in RYC, introducing elevated comorbidity rates in this study. On the other hand, longitudinal findings in the BEIP revealed that placement disruptions predicted psychopathology, rather than vice versa [72], which may also apply to adolescents in RYC. Indeed, the participants in our study had multiple placement disruptions and high ages at first placement, both key risk factors of emotional and behavioral problems in looked-after children [74]. Further, developmental changes from childhood to adolescence, i.e., higher prevalence rates of emotional, behavioral and substance use disorders in adolescence [14, 67], may contribute to the higher psychiatric morbidity in this study compared to studies of younger children. In any case, the findings demonstrate high levels of comorbidity and additional psychosocial burdens for adolescents with RAD and DSED in RYC settings. Of note, even though most adolescents with RAD or DSED qualified for at least one additional psychiatric disorder, no single diagnostic category was present in more than half of those with RAD or DSED, and only a minority of adolescents with the other disorders had comorbid RAD or DSED, supporting previous findings of the discriminant validity of RAD and DSED in adolescence [21].

### RAD

Finding that both emotional and behavioral problems may co-occur with RAD and be associated with RAD symptom load in adolescence is concordant with previous findings among school-aged children and early adolescents [26, 41–43]. However, this result contradicts that of studies among pre-schoolers, which report that RAD is associated with more emotional problems and not with more behavioral problems [39, 40, 75]. Although there is a possibility of type-II errors where observed differences are not statistically significant due to small sample sizes, the above studies of pre-schoolers used dimensional measures of psychopathology, thereby eliminating potential type-II errors due to reduced power by categorical measures. Therefore, rather than having predominantly methodological explanations, the differences in the findings between the associations of RAD in pre-schoolers and adolescents may be due to real developmental changes from childhood to adolescence, and the added risk from many placement disruptions and high age at the first placement.

Although depression and anxiety were the most common comorbid disorders among adolescents with RAD, CD/ODD was nearly as common and present in 40% of those with a RAD diagnosis. This high comorbidity rate may shed light on the conceptual confusion pertaining to older children and adolescents, whose conduct problems may be misinterpreted as RAD [9, 76]. Importantly, our findings clearly demonstrate that while many with RAD had co-occurring CD/ODD, most adolescents with RAD did not. Moreover, despite the high-risk nature of the sample, only a small minority (14%) of all adolescents with CD/ODD had a co-occurring RAD diagnosis. Further, although RAD symptom load was associated with the dimensional measure of aggressive behavior, illustrating that adolescents with RAD may have additional behavioral problems, neither the categorical measures of CD/ODD or contact with the police, nor the dimensional measure of rule-breaking behavior was associated with RAD. Therefore, although both conduct problems and RAD are associated with maltreatment and neglect and are malleable by caregiver behavior [39, 72], conduct problems in individuals with histories of maltreatment and neglect are not equivalent to RAD and should not be interpreted as such.

The lack of increased odds of most other forms of psychopathology with a RAD diagnosis, as opposed to not having RAD, must be understood in light of the high-risk nature of the sample, with a very high psychiatric morbidity also among the adolescents without RAD. Further, the reduced statistical power caused by dichotomizing RAD symptoms into a RAD diagnosis (present/absent) may partially explain a loss of statistical significance from the dimensional measure of RAD symptom load to the categorical RAD diagnosis. However, such a trend was not obvious where a categorical approach to co-occurring psychopathology and psychosocial problems was used. Indeed, we see the opposite tendency for the odds of co-occurring CD/ODD and ADHD, with lower p-values for a RAD diagnosis than for RAD symptom load. Furthermore, although in the dimensional approach to other forms of psychopathology we see the expected loss of statistical significance by categorizing RAD symptoms into a RAD diagnosis, we note a lack of clinically significant regression coefficients for a RAD diagnosis (except for the association with CBCL ‘withdrawn/depressed’), contrary to the RAD symptom load, illustrating that different approaches to RAD may reveal different results regardless of statistical power. One reason for this may be that the RAD diagnosis reflects more than a numerical cut-off level of RAD symptoms, as the diagnostic criteria require the presence of certain symptom clusters classified under A criteria (minimal comfort seeking/response) and B criteria (emotional dysregulation and limited emotional responsiveness) [4]. In a study of foster youth, self-reported potentially traumatic events were associated with B criteria, not with A criteria of DSM-5 RAD [77]. Possibly, the A and B criteria also differ in their associations with other psychopathology, potentially impacting our results.

Interestingly, in this high-risk sample, the sole association between a RAD diagnosis and dimensional measures of psychopathology was with the CBCL withdrawn/depressed scale, mirroring findings among institutionalized pre-schoolers, where an observational measure of RAD was strongly related to the CBCL scales withdrawn/depressed and somatic complaints—though only weakly to a total score of emotional problems (internalizing score)—and were not associated with behavioral problems [75]. Due to multiple hypotheses and p-values being between .01 and .05, the positive associations between RAD symptom load and the categorical measures of depression and self-harm, and between a RAD diagnosis and suicidal thoughts, must be interpreted with caution. However, the statistically convincing associations between a RAD diagnosis and the CBCL withdrawn/depressed scale, and between RAD symptom load and most CBCL syndrome scales, demonstrate the importance of assessing emotional problems in adolescents with RAD.

### DSED

The prevalence rates of ADHD and CD/ODD in adolescents with DSED resemble findings in preschool and school-aged children with signs of DSED, including home-reared [11] and post-institutionally adopted [44] children. Concordant with findings in preschool, school age and young adulthood [11, 19, 45, 47], we found DSED in adolescence to be associated with ADHD. Although we failed to reveal associations between DSED and categorical emotional disorders, the most frequently co-occurring disorders among adolescents with DSED were depression and anxiety, each present in over half of those with a DSED diagnosis. Emotional problems were more prevalent among adolescents with DSED in this sample than reports of post-institutionalized adopted school children [44], possibly reflecting developmental differences such as increasing emotional problems in adolescence [2] or factors related to the care context [74], as discussed above in relation to RAD. Further, three of the findings—that suicidal thoughts occurred more frequently in adolescents with DSED than in those without, that half of the adolescents with a DSED diagnosis reported previous suicidal attempts, and that adolescents with a DSED diagnosis were more prone to intentionally using substances for mood improvement—underscore the importance of assessing emotional problems, including suicidality and emotion regulation from substance use, in adolescents with DSED.

Due to multiple hypotheses and p-values being between .01 and .05, the associations between DSED in adolescence and the categorical measures of ADHD, any comorbid disorder, suicidal thoughts and substance use for mood improvement must be interpreted with caution. Nevertheless, as for RAD, the few and weak associations between a DSED diagnosis and the categorical measures of co-occurring psychopathology may be masked by the high-risk nature of the comparison group with a very high psychiatric morbidity in adolescents without DSED. Even so, all the investigated disorders and psychosocial problems were numerically more prevalent among adolescents with DSED diagnosis than those without. Further, as the dimensional approach to co-occurring psychopathology revealed strong associations between a DSED diagnosis and both emotional and behavioral problems, while the categorical approach did not, it seems plausible that results regarding the latter are subject to type-II error due to the reduced power of the categorical dependent variables.

The positive associations between DSED symptom load and all but two CBCL syndrome scales (withdrawn/depressed and somatic complaints) cohere with findings in other non-institutionalized adolescents exposed to in-family maltreatment, where DSED symptoms were strongly associated with all CBCL syndrome scales except the withdrawn/depressed and somatic complaint scales [46]. The overall finding that DSED in adolescence is associated with both emotional and behavioral problems is also in line with some results from studies of younger children [36, 39].

### Strengths and limitations

The use of in-depth semi-structured psychiatric interviews in a national and comparatively large sample of high-risk adolescents constitutes a clear strength. Further, the combined use of self- and caregiver reports for other psychopathology and psychosocial problems reduced the risk of common rater bias. However, we acknowledge some limitations. Our assessment of RAD and DSED was limited to caregiver (primary contact) information. Although a caregiver-informed approach to RAD and DSED is common in research [2, 23, 24], clinical recommendations entail a multi-method approach, including observational assessments [13, 65]. Both a risk of over-identification [78] and under-identification [12] have been demonstrated in caregiver reports of RAD and DSED. However, caregiver assessments of RAD and DSED have also been found to converge with observational measures [13, 19, 22, 43], lending support to our findings. A related limitation for RAD, but not for DSED, is uncertainty as to whether aberrant attachment behavior registered by the primary contacts in the RYC was representative of the adolescents’ attachment behavior toward previous caregivers. Previous findings of the trans-relational nature of RAD [43] support the suitability of our approach. Further, for the DSM-5 RAD A criterion, we only had available information on the adolescents’ comfort-seeking behavior and no information on their response to comfort. This may have influenced our results by deflating the number of RAD symptoms in the measure of symptom load and inflating the number of participants with a RAD diagnosis. Additionally, we were only able to substantiate, not document with certainty, the DSM-5 criteria of early exposure to extremely insufficient care and the presence of RAD symptoms prior to age 5, possibly inflating our diagnostics of RAD and DSED. However, careful measures were taken to minimize the risk of over-diagnosing RAD and DSED, and the prevalence rates of RAD and DSED herein are concordant with the findings in foster children in Norway [33]. Thus, we consider the risk of overdiagnosis to be limited.

Due to developmental changes and heterotypic continuity of disorders and symptoms, the rates of co-occurrence and the degree of associations reported herein cannot necessarily be ascribed to other age groups. Further, the prevalence rates of comorbid disorders and psychosocial problems are likely to be context dependent, and may therefore differ for adolescents in non-RYC settings—such as adolescents with early placement in well-functioning and lasting foster/adoptive homes or adolescents placed in larger-sized or less developmentally supportive RYCs—and those in other countries. Because adolescent behavioral problems and drug use are more frequently cited reasons for placement in RYC than in foster care [54], we would expect foster-placed adolescents with RAD and DSED to have somewhat lower co-occurrence of behavioral problems and drug use than adolescents with RAD and DSED in RYC.

### Clinical implications

As this is the first in-depth and multi-approach investigation of co-occurring psychopathology and psychosocial problems among adolescents with RAD and DSED, the clinical value is presumably high. Because other psychiatric disorders and psychosocial problems frequently co-occur with RAD and DSED in adolescence, all adolescents with RAD or DSED symptoms or diagnoses should receive comprehensive psychiatric assessment in accordance with the practice parameter [65]. Clinicians should, in their assessments of adolescents with RAD or DSED, systematically consider possible comorbid emotional and behavioral disorders as well as related psychosocial problems, including suicidality, bullying experience, juridical offenses, sexual activity and substance use. Because disclosing such problems may provoke feelings of shame and taboo, adolescents may not spontaneously present them in conversation or general assessment. However, becoming aware of these additional psychosocial problems might impact the overall understanding of the adolescent’s daily challenges and might be crucial in terms of offering adequate treatment and support. Our findings underline the importance of permitting diagnostic comorbidity so that all aspects of an individual’s mental health problems may be incorporated into a comprehensive understanding of what support and treatment are needed. This is contrary to the general medical principle of combining symptoms to a minimum number of diagnoses. We maintain, however, that the discriminant validity demonstrated for RAD and DSED in previous studies, combined with this and other studies demonstrating the clinically important ramifications of early maltreatment and neglect, imply that clinicians should seek to grasp the full complexity rather than simplify their understanding in the assessment and treatment of these high-risk individuals.

## Conclusion

Most adolescents with RAD or DSED disorders or symptoms have additional psychiatric disorders and psychosocial problems of an emotional and/or behavioral nature, warranting easy access to high-quality psychiatric health care, including a comprehensive psychiatric assessment where comorbidity is acknowledged, and treatment plans are adjusted accordingly.

## Electronic supplementary material

Below is the link to the electronic supplementary material.

Supplementary material 1 (DOCX 70 kb)

## Data Availability

Access to data by project manager Nanna S. Kayed.
