# Supplementary material for: Reactive attachment disorder and disinhibited social engagement disorder in adolescence: co-occurring psychopathology and psychosocial problems
Source: Eur Child Adolesc Psychiatry. 2020 Nov 13;31(1):85–98. doi: 10.1007/s00787-020-01673-7 (PMC8816327; doi:10.1007/s00787-020-01673-7)
Supplement: Supplementary file 1 — Supplementary material 1 (DOCX 70 kb) [file 787_2020_1673_MOESM1_ESM.docx]

# Online Resource 1

**Article**: Reactive Attachment Disorder and Disinhibited Social Engagement Disorder in Adolescence: Co-occurring Disorders and Psychosocial Problems

**Journal:** Journal of European Child and Adolescent Psychiatry

Figure S1. Participant flowchart

19 primary contacts did not complete the PAPA

Participants in the main study

N = 400 (Response rate 67%)

Multiple imputation
complete dataset

N = 381

59 adolescents did not complete the
CAPA

Participants with completed
PAPA and CAPA

N = 322 (81% of 400)

Participants with completed PAPA yielding information about
RAD and DSED

N = 381 (95% of 400 participants)

Note. *CAPA* child and adolescent psychiatric assessment; *DSED* disinhibited social engagement disorder; *PAPA* preschool age psychiatric assessment; *RAD* reactive attachment disorder.
